# Knowledge, assessment and treatment of molar incisor hypomineralisation (MIH) among German dentists

**DOI:** 10.1007/s00784-025-06249-w

**Published:** 2025-03-07

**Authors:** Carla Ostermann, Christian Splieth, Mohammad Alkilzy

**Affiliations:** Department of Pediatric Dentistry, University of Greifswald, Greifswald, Germany Fleischmannstr. 42, 17475

**Keywords:** MIH, Molar incisor hypomineralisation, Knowledge, Perception, Questionnaire

## Abstract

**Objectives:**

This study investigated the knowledge, perceptions, and clinical practices of German dentists regarding molar incisor hypomineralisation (MIH), a significant issue in paediatric dentistry. To date, no study has examined the knowledge and experience of German dentists with MIH.

**Subjects and methods:**

A digital questionnaire comprising 25 items was designed to assess German dentists’ understanding of MIH. The questionnaire was available online via Survio.de from March to June 2023. The responses were entered anonymously into an Excel spreadsheet and analysed using SPSS 29.0.

**Results:**

625 dentists were surveyed, and 517 questionnaires were evaluated. 99.2% of respondents treated patients with MIH, and 92.5% considered it a significant clinical problem. Commonly observed clinical presentations included yellow/brown defects (81%), enamel loss (46%) and white defects (36.4%). 68.6% of respondents noted an increase in MIH prevalence. Treatment barriers included child behaviour (44.7%), difficulties with local anaesthesia (22.7%), and extended treatment duration (15.3%). Most dentists (77.8%) expressed a desire for further training on MIH.

**Conclusions:**

The data indicated that dentists’ MIH management was influenced by specialisation and diagnostic confidence. Despite basic knowledge and experience, many respondents expressed a need for further education. Age-related biases and limited awareness of new techniques highlight the need for further research.

**Clinical relevance:**

Dentists must receive comprehensive training to enable them to diagnose and treat patients promptly. This should include the development of updated courses, which should be aligned with international guidelines.

## Introduction

Molar incisor hypomineralisation (MIH) is a pressing concern in paediatric dentistry worldwide. Initially defined by Weerheijm et al. in 2001 [1], MIH has been identified under various nomenclatures. The condition poses considerable treatment challenges, impacting both aesthetics and function, rendering affected teeth more prone to caries and rapid breakdown [2]. The quality of life for those affected by MIH is significantly impaired, underscoring the importance of continued research and clinical focus [3].

While the aetiology and pathogenesis are still under investigation, established diagnostic criteria aid in the formulation of appropriate therapeutic recommendations [4]. The European Academy of Paediatric Dentistry (EAPD) in 2003 defined diagnostic criteria for MIH, including clearly delineated opacities, sensitivity, enamel breakdown, atypical restorations, and tooth extraction [5].

The prevalence of MIH varies significantly, ranging from 3 to 44% and 0.5–40%, depending on the study and country [6–9]. A meta-analysis of pooled data estimated a global MIH prevalence of 13.5%, with no significant difference between genders [8–10]. In Germany, the prevalence of MIH was documented for the first time in the 5th German Oral Health Study 2018, which found that 28.7% of 12-year-olds had at least one first permanent molar with MIH [11].

The multifactorial aetiology of MIH is globally acknowledged, likely driven by gene-environment interactions [12]. Key risk factors during critical tooth development stages include prenatal and childhood exposures such as maternal illness, premature birth, low birth weight [13–15], prenatal lead exposure [15], and antibiotic use [13, 14, 16]. Genetic predispositions, potentially influenced by environmental factors, have been indicated by family and twin studies [7, 17, 18], but further research is needed to fully elucidate MIH’s origins [19].

These hypomineralisation defects result in reduced mineralisation and increased protein and water content, making the enamel porous and discoloured, which can lead to higher susceptibility to caries, sensitivity, and breakdown [13, 20, 21]. The likelihood of caries in these teeth can be up to 4.6 times greater compared to normal teeth [22]. Such teeth, particularly molars, are more vulnerable to masticatory damage than incisors [23] and display heightened sensitivity to thermal, mechanical, and air stimuli [1, 24]. Bacterial permeation through defective enamel may cause subclinical pulp inflammation and abnormal pulpal nerve density [25, 26]. Early identification of these defects facilitates timely preventative and restorative treatments to mitigate caries, pulp inflammation, and hypersensitivity, while also addressing aesthetic and psychosocial issues [27, 28]. Proactive management includes optimising oral hygiene, dietary counselling, sensitivity management, and remineralisation strategies [28, 29]. However, hypersensitivity may complicate oral hygiene and make dental treatments difficult [22, 27, 28, 30, 31], potentially impacting the child’s quality of life and contributing to dental fear and treatment fatigue [32].

The aim of the study was to assess the general level of knowledge of German dentists regarding MIH, focusing on aspects such as aetiology, diagnosis, treatment approaches, observed clinical presentations, and treatment barriers. The research sought to explore dentists’ preferences for additional training in MIH and their confidence levels in diagnosing MIH cases to adapt the training offers at the universities or dental practices.

## Subjects and methods

The questionnaire was based on the one developed by Weerheijm et al. [23]. Subsequent modifications were implemented by an Australian research group [33]. These alterations included additional questions concerning clinical problems, aetiology, and restorative care, thus expanding the scope of the original questionnaire to better assess dentists’ knowledge, perceptions, and clinical experiences regarding MIH [33]. Prior to data collection and analysis, ethical approval was obtained from the ethics committee of Medicine University Greifswald and registered under number BB 067/23.

The questionnaire was divided into two primary sections. The first gathered demographic data from the participating dentists, such as age, gender, professional experience, dental specialisation, and familiarity with MIH in their practice. The second focused on assessing the dentists’ overall proficiency in MIH, covering its aetiology, treatment strategies, clinical presentation, challenges, and interest in additional training.

The survey consisted of 25 items and took about five to ten minutes to complete. It was distributed online via ‘Survio.de,’ with a link provided to participants and a cover letter detailing the study’s objectives, voluntary participation, and confidentiality assurances. To maximise participation, the questionnaire was shared on various dental websites and portals, and several dental associations were contacted to include it in their newsletters. The questionnaire was promoted on the websites of the Landeszahnärztekammern in Baden-Württemberg and Brandenburg, as well as the Kassenzahnärztliche Vereinigung Westfalen-Lippe, Mecklenburg-Vorpommern, Sachsen-Anhalt, Berlin, and Baden-Württemberg. Additionally, it was posted in the “Dentalfamilie” Facebook group and featured in the “Zahnärztliche Nachrichten Sachsen-Anhalt” and “Fan (Fachassistenznews)” publications. The survey was launched on March 8, 2023, and remained available online until June 30, 2023.

No age or gender limitations were set for participation in the questionnaire. The collected data was verified for completeness, compiled in Excel, and handled with strict confidentiality. The information was anonymised and analysed comprehensively alongside data from all participating dentists to ensure individual responses could not be identified.

SPSS 29.0 [SPSS for Windows, version 29.0, SPSS Inc., Chicago, IL, USA] was used for analysis. The analysis allowed comparisons between dentists based on the distribution of selected biographical variables, such as age and workplace. Descriptive statistics were generated, and the Chi-square test was used for nominal or ordinal variables. The significance level was set at *p* = 0.05. Effect sizes were measured using the Phi (φ) or Cramer V coefficients. Given non-homogeneous variance and non-normal distribution across groups, a non-parametric test (Kruskal-Wallis test) examined the frequency of MIH treatment over the past four weeks, considering age and work settings.

## Results

Out of the 625 received filled questionnaires, 108 were incomplete. Instances of duplicate responses and inconsistencies in binary questions led to the removal of some data records to ensure data quality. Despite incompleteness, data records with partially answered questions were retained for statistical analysis. Ultimately, 517 questionnaires were included in the dataset, resulting in a sample size of *N* = 517.

The analysis included general demographic parameters such as gender, age, specialisation, and experience with MIH. Of the 517 respondents, 72.1% identified as female, 27.5% as male, and 0.4% as mixed gender. Age distribution showed 5.4% were under 30 years, 24.6% between 30 and 39, 26.5% between 40 and 49, and 43.5% were aged 50 or older. Approximately 37.5% reported specialised dental training, including fields such as paediatric dentistry, orthodontics, periodontics, oral surgery, and prosthodontics. The general parameters are presented in Table 1. Nearly all surveyed dentists (99.2%) reported providing care to MIH patients in their clinical practice.

**Table 1:** Characteristics of the study sample regarding the general parameters of age group, workplace subdivided for clinical management or no treatment of MIH patients

	Patients with MIH		No patients with MIH		Total result	
	*N*	%	*N*	%	*N*	%
Age < 30 years	26	5.1	1	0.2	27	5.3
University	4	0.8	0	0	4	0.8
Private practice	15	2.9	1	0.2	16	3.1
Public health service	2	0.4	0	0	2	0.4
Others	6	1.2	0	0	6	1.2
Age 30–39 years	125	24.3	1	0.2	126	24.5
University	4	0.8	0	0	4	0.8
Private practice	94	18.3	1	0.2	95	18.5
Public health service	7	1.4	0	0	7	1.4
Others	21	4.1	0	0	21	4.1
Age 40–49 years	135	26.3	2	0.4	137	26.7
University	1	0.2	0	0	1	0.2
Private practice	119	23.2	0	0	119	23.2
Public health service	4	0.8	2	0.4	6	1.2
Others	13	2.5	1	0.2	14	2.7
Age ≥ 50 years	224	43.6	0	0	224	43.6
University	4	0.8	0	0	4	0.8
Private practice	208	40.5	0	0	208	40.5
Public health service	5	1	0	0	5	1
Others	9	1.8	0	0	9	1.8
Total result	510	99.2	4	0.8	514	100

*N* = 514

**Fig. 1:**
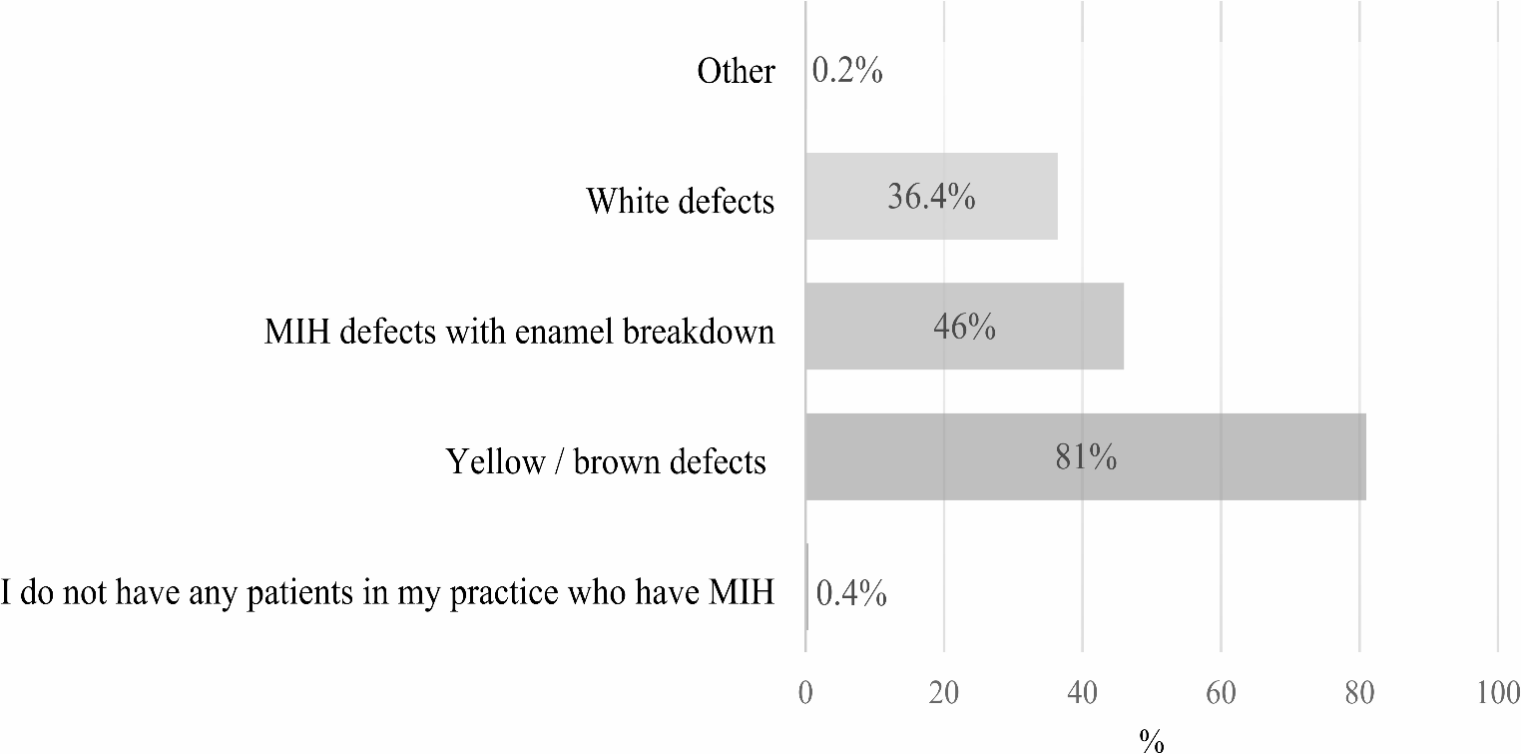
Most frequently MIH presentations observed by the study sample (*N* = 511) allowing multiple answers

Among the 514 dentists treating MIH patients, 31.6% expressed high confidence in diagnosing the condition, 59.4% felt confident, and 9% were uncertain, with none reporting high uncertainty. A statistically significant association was found between diagnostic confidence and specialisation (χ²(3) = 21.034, *p* < 0.001, φ = 0.202). Figure 1 shows the most common clinical presentations of MIH observed by the dentists surveyed.

Approximately half of the surveyed dentists (49.9%) observed similar defects in permanent teeth beyond the incisors and first molars. Furthermore, 60% noted demarcated hypomineralised defects in primary teeth, primarily in molars (239 instances), incisors (31 instances), and canines (23 instances). A majority, 68.6% (352 dentists), reported an increasing prevalence of MIH in their regions, whereas 10.7% (55 dentists) did not observe this trend, and 20.7% (106 dentists) were uncertain. There was a notable association between dentists who treat patients with MIH in their practices and their perception of a rise in MIH prevalence (χ²(3) = 257.956, *p* < 0.001, φ = 0.706). Additionally, a significant correlation occurred between the diagnostic confidence of surveyed dentists and their belief in the upsurge of MIH prevalence (χ²(9) = 37.603, *p* < 0.001, Cramer V = 0.156).

The investigation into the frequency of MIH-diagnosed patient encounters over the past four weeks considered dentists’ age and workplace location, using median values to account for statistical outliers. Table 2 illustrates both median and mean values, which differ due to outliers.

**Table 2:** Numbers of patients treated with MIH in the past 4 weeks in relation to the age group of the treating dentists

Age groups	Median value	Mean value	*N*
< 30 years	1.5	3.04	28
30–39 years	2.0	5.46	127
40–49 years	3.0	13.71	137
≥ 50 years	3.0	6.57	225
Total result	3.0	8.00	517

*N* = 517, Kruskal-Wallis Test, independent samples, asymptotic, 2-sided: *p* = 0.016

The analysis of age groups revealed significant differences (*p* = 0.016, Kruskal-Wallis Test), indicating variations in variances and non-normal distributions among specified groups. Noteworthy differences among age categories were observed, with individuals under 30 differing significantly from those over 50 (*p* = 0.018) and 40–49 (*p* = 0.011). Additionally, a significant difference was observed between the 30–39 and 40–49 age groups (*p* = 0.030).

The majority of participants (478 out of 517 individuals) regarded teeth affected by MIH as a clinical problem. A significant association was found between the diagnostic confidence of the dentists and their perception of MIH as a clinical issue (χ²(3) = 12.467, *p* = 0.006, φ = 0.155).

A definitive statement regarding regional prevalence was refrained from by 11% of participants (57 individuals). Additionally, 27.7% (143 individuals) perceived the regional prevalence as low (less than 5%), while 35.4% (183 individuals) estimated it to be between 5 and 10%. Moreover, 20.3% (105 individuals) indicated a prevalence of 10–20%, and 5.6% (57 individuals) suggested a prevalence exceeding 20%. The arithmetic mean was 5–10%. A statistically significant association was observed between regional prevalence estimation and diagnostic certainty (χ²(12) = 40.085, *p* < 0.001, Cramer V = 0.161).

**Fig. 2:**
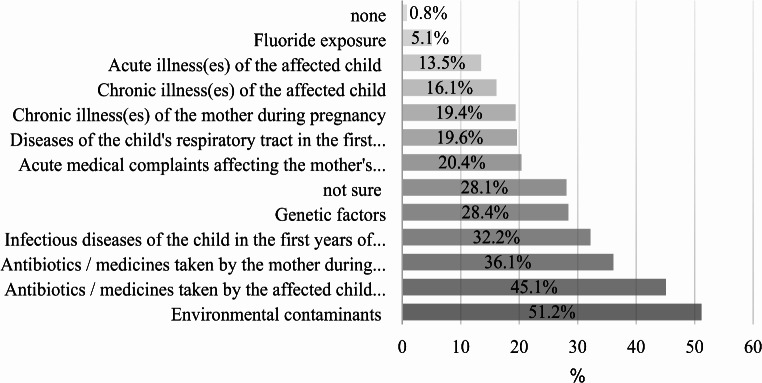
Distribution (%) of etiological factors associated with MIH by respondent dentists (*N* = 490)

Multiple potential causes of MIH were identified, with “Environmental contaminants” being the most commonly cited factor at 51.2%. There was a notable uncertainty among participants (28.1%). The remaining values and specific factors can be referenced in Fig. 2. Respondents also cited various additional factors such as environmental exposure to plastic, Bisphenol A, dietary habits of both child and mother, maternal veganism, and calcium, vitamin D, and K deficiencies. Additionally, factors like birth circumstances, medications during childbirth, maternal age, extended breastfeeding, maternal dental fillings, and parental drug use were identified by respondents as potential causal elements for MIH.

The survey revealed varied responses regarding the timing of enamel formation disruption. The most frequently cited period was “within the first year of life” (37.8%), followed by “during the third trimester of pregnancy” (35.7%), and “not sure” (30.8%). About 25.9% of dentists speculated that damage could occur between the first and second years of life, while 16.6% believed it might happen during the second trimester of pregnancy. The lowest perceived risk (10.3%) was associated with the first trimester of pregnancy. Additionally, 11.7% considered the period between the second and third years of life as a potential timeframe for ameloblast damage.

Among surveyed dentists, 86.7% (448 individuals) affirmed familiarity with treating children with MIH, while 13.3% (69 individuals) responded negatively. Statistical analysis revealed a significant association between dentists’ familiarity with MIH treatment and their age (χ²(3) = 14.568, *p* = 0.002, φ = 0.168), as well as completion of specialisation (χ²(1) = 6.980, *p* = 0.008, φ = 0.116).

Out of the surveyed dentists, 36.5% (155 individuals) would have referred children with MIH to a paediatric dentist, while 45.2% (192 individuals) would not, and 18.4% (78 individuals) were unsure. Paediatric dental specialists were excluded from this analysis. A significant association was found between familiarity with MIH treatment and the decision to refer to a paediatric dentist (χ²(2) = 19.750, *p* < 0.001, φ = 0.195).

**Fig. 3:**
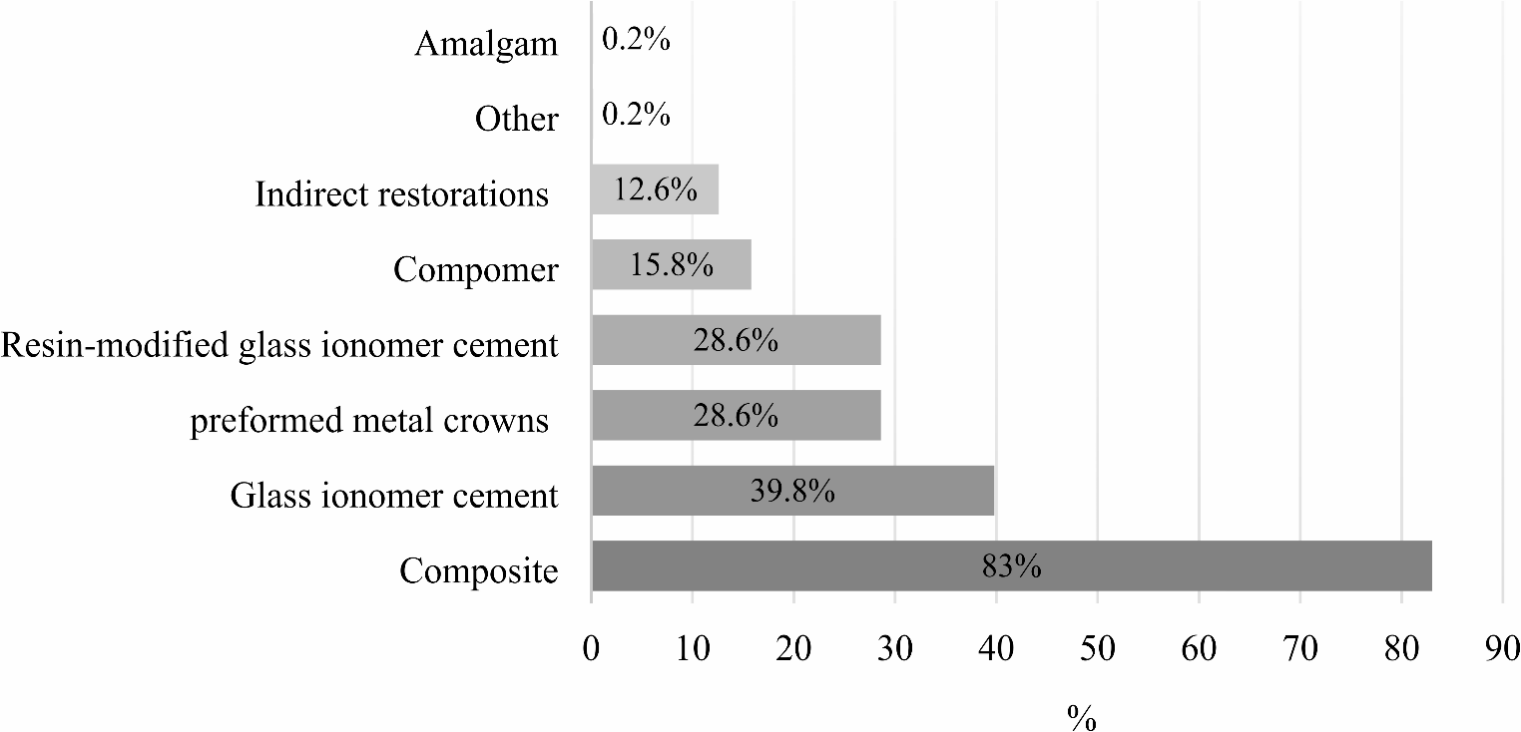
Distribution (%) of restorative materials used by surveyed dentists for MIH teeth (*N* = 493)

The survey highlighted diverse preferences in treatment materials for MIH, as shown in Fig. 3. “Composite” was the most selected material (83%), followed by “Glass ionomer cement” (39.8%), “Resin-modified glass ionomer cement,” and “Stainless-steel crowns” (both 28.6%). A significant association was found between dentists’ diagnostic confidence in MIH and the use of stainless-steel crowns (χ²(3) = 20.055, *p* < 0.001, φ = 0.197). Mentioned “other” materials included fluoride varnishes, fissure sealants, ICON, Fuji Triage, and high-dose daily home fluoride application. Dentists working at universities showed a higher preference for glass ionomer cement (χ²(1) = 12.107, *p* < 0.001, φ = 0.153), stainless-steel crowns (χ²(1) = 21.620, *p* < 0.001, φ = 0.204), and indirect restorations (χ²(1) = 8.604, *p* = 0.003, φ = 0.129) in MIH treatment compared to other workplace settings.

A substantial proportion of dentists (64.8%) viewed tooth extraction as a viable solution for MIH, with 87.8% preferring to seek orthodontic consultation beforehand. Dentists outlined various scenarios where extraction might be considered, including severely damaged teeth, space limitations, the presence of third molar, patient discomfort, non-compliance, and cases where crowning for long-term retention is impractical.

**Fig. 4:**
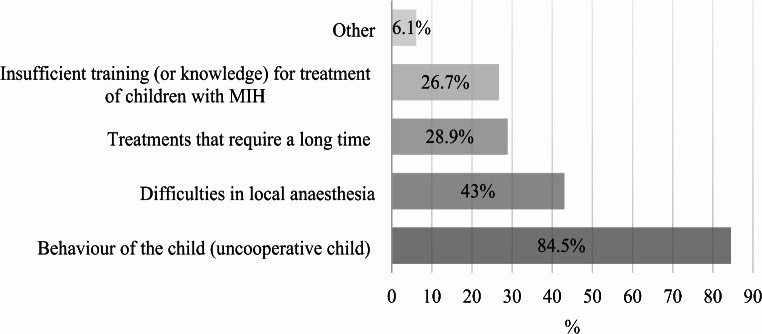
Distribution (%) of the barriers to treating MIH patients identified by the surveyed dentists (*N* = 509)

The challenges perceived by the treating dentists during treatment are shown in Fig. 4.

440 individuals (85.1%) reported receiving information about MIH, while 77 individuals (14.9%) did not. The sources of information included dental journals (458 responses, 89.5%), further education (308 responses, 60.2%), the internet (222 responses, 43.4%), books (64 responses, 12.5%), brochures (51 responses, 10.0%), and other sources (31 responses, 6.1%), such as professional development courses, conferences, seminars, webinars, lectures, collegial exchange, podcasts, PubMed, and direct communication with universities. The majority of respondents (77.8%) expressed interest in receiving further education on MIH.

## Discussion

This study provides novel insights into the knowledge, clinical experience, and perception of MIH among German dentists, identifying key challenges and barriers in MIH treatment that could be addressed through targeted education and training. By illuminating these issues, the research raised awareness of interventions to close knowledge gaps, improve treatment efficiency, and enhance clinical outcomes. The study highlights the awareness of MIH and the challenges faced by the dental community. It also demonstrates a clear need for continuing education that would contribute to the development of expertise. Similarly, in Greece, Seremidi et al. emphasised the necessity for clear clinical guidelines, as Greek dentists reported variability in their diagnostic approaches to MIH. These findings align with those of the study and support the call for standardised diagnostic guidelines on a European and international level [34].

The demographic profile of the surveyed dentists, predominantly female (72.1%) and including a significant number of individuals over the age of 50 (43.5%), mirrors global trends in the feminisation and aging of the dental profession [35, 36]. The demographic composition of the sample might have influenced perceptions of MIH and treatment preferences, potentially introducing bias if respondents included a subgroup with a particular interest in MIH. The prevalence of older, experienced dentists in the sample might have led to a bias towards traditional, conservative treatment approaches. These findings underscore the importance of examining how dentists’ demographic characteristics shape clinical practice.

The study demonstrated a high level of engagement among dentists in the treatment of MIH, with 99.2% of the surveyed dentists reporting the presence of MIH patients in their practice. This result suggests that MIH is a common clinical problem among dentists, consistent with the findings of other studies [37–41]. However, it is possible that the high number of dentists treating MIH reflects the fact that the survey respondents were primarily dentists who were interested in and familiar with MIH. Moreover, the confidence levels in diagnosing MIH were generally high, with most dentists reporting certainty (59.4%) or very high certainty (31.6%). The relatively low number of dentists who reported uncertainty (9%) suggests an overall self-assuredness in managing MIH cases. These confidence levels are similar to those reported in the United Kingdom in 2016, where most respondents felt certain or very certain about diagnosing this condition [42].

As can be seen in Fig. 1, enamel breakdown was observed as a more common clinical presentation than white defects among the dentists surveyed. Other studies have noted a higher prevalence of white demarcated opacities compared to enamel breakdown [40]. The discrepancy might suggest varying levels of examination skills among dentists, particularly in identifying less obvious defects such as subtle white patches. This indicates a potential need for enhanced training and education for dentists in Germany to improve diagnostic accuracy across different manifestations of MIH. Nearly half of the dentists reported defects in permanent teeth beyond incisors and first molars, and 60% observed demarcated hypomineralised defects in primary teeth, highlighting the need for customised strategies for both primary and permanent dentitions.

Responses to a survey concerning the timing of enamel disruption revealed a range of perspectives. The most commonly identified timeframe was “within the first year of life,” with 194 respondents (37.8%) indicating early onset. Similarly, dentists in Western North America predominantly suspected ameloblast damage during this period (35%) [43]. Additionally, 183 participants (35.7%) selected “during the third trimester of pregnancy,” suggesting a prenatal influence. The responses also revealed considerable uncertainty: 158 respondents (30.8%) were unsure of the time, reflecting the complexity of determining the time of origin. Environmental contaminants, medications, and genetic factors were among the potential causes mentioned. Although some literature points to a connection between plastic softeners and MIH [44, 45], clear correlations remain elusive. Links were also observed between preterm births, low birth weights [46], maternal alcohol consumption [47], and the occurrence of MIH. The role of vitamin D deficiency as an etiological factor is disputed [48, 49], while some research implicates environmental contaminants [45]. Recent studies suggest a genetic basis, emphasising that perinatal and postnatal influences on MIH are more significant than prenatal factors [50]. Further studies from various countries showed that dentists often regard the aetiology of MIH as multifactorial [33, 37, 51–53]. These observations underscore MIH’s complex nature and the need for further research to develop effective prevention and treatment strategies.

The data provided insights into dentists’ perceptions of regional MIH prevalence, with an estimated mean of 5–10%. A significant association was found between regional prevalence estimates and diagnostic certainty, indicating that confidence in diagnosis influences how dentists assess MIH prevalence. International estimates vary widely [6–9], highlighting the need for continuous education to ensure accurate knowledge and understanding. The variability suggests that geography, demographics, and environmental factors may influence regional assessments.

The survey findings on familiarity with treating children with MIH showed that most dentists (86%) were confident in managing MIH, a figure consistent with surveys from Oslo (68%) [54] and Mexico (61%) [40]. Responses also revealed varied opinions regarding the referral of MIH cases to paediatric dentists; while 36% indicated a readiness to refer, 45% preferred managing such cases independently, and 18% were uncertain. In Oslo, only 27.8% expressed readiness to refer [54], compared to 59% in Mexico [40]. The correlation between familiarity with MIH and referral tendencies suggests that increased knowledge and experience enhance recognition of when specialised care is necessary, highlighting the potential for targeted educational programs to improve referral practices.

As can be seen in Fig. 3, composites were the most commonly used treatment materials, aligning with EAPD recommendations that emphasise composites for aesthetic and functional benefits, especially in visible areas [6]. Glass ionomers are preferred for their fluoride release and remineralisation properties [55], while preformed metal crowns are used for heavily compromised molars due to their durability [56]. Egyptian dentists, similarly to the findings of this study, favoured composites (74%), resin-modified glass ionomer cement (48.2%), and preformed crowns (41.6%) for MIH treatment [57]. Treatment choices should consider patient age, tooth type (incisor or molar), and MIH severity [6]. The preference for composites and the option of tooth extraction, favoured by 64.8% of surveyed dentists, may reflect an age-related bias. A large portion of respondents are over 50, potentially favouring conventional over current minimally invasive practices. Most respondents (87.8%) would have consulted orthodontists before extraction, underscoring the importance of interdisciplinary collaboration in MIH treatment and emphasising patient-centred care focused on both functional and aesthetic outcomes.

Inadequate training in treating MIH was highlighted by 14.1% of respondents, emphasising the need for better dental education. Courses for general dentists and enhanced university-level education could be instrumental in addressing this issue. Additionally, the lack of sufficient compensation for treatments, particularly those not covered by insurance, demotivates dentists. Respondents also noted significant concerns such as the poor durability of dental materials and increased sensitivity in affected children. In Mexico, 86% of dentists reported concerns over limited MIH training, 43% identified uncooperative child behaviour as a treatment hurdle, and 16% noted the extended duration of treatment as problematic [40]. In Kuwait, the primary barriers were similar [43]. North American practitioners highlighted the lengthy time required for restorative treatments as a significant challenge [51], while Greek dentists reported that children with MIH are 2 to 5.5 times more likely to experience difficulties in achieving adequate anaesthesia and face issues with hypersensitivity [34]. These findings underscore the complex array of challenges in treating children with MIH, which include behavioural, procedural, educational, financial, societal, and material-related factors.

The majority of respondents (85.1%) reported receiving information about MIH from various sources, predominantly dental journals. In Mexico, the most frequently cited source was the Internet (36%), followed by dental courses (33%) and professional journals (28%) [40]. In Saudi Arabia, dental journals were the primary source of information for 40% of dentists [38], whereas in Kuwait, the Internet was the main source for 37% of surveyed dentists [43]. This indicates a varied reliance on both traditional and digital channels for obtaining information. Given the prevalence of dental journals as an information source among German dentists, publishing new MIH findings in these journals could significantly broaden their impact within the dental community. A substantial proportion of dental professionals (77.8%) indicated their readiness to pursue additional education on MIH, underscoring their commitment to expanding their understanding and staying updated with advancements in this field. This inclination is consistent with international trends, as evidenced by similar findings in Mexico where 86% of dentists surveyed expressed an interest in further MIH education [40]. This willingness for continuing education reflects the professionals’ commitment to staying abreast of the latest developments and treatment methodologies, potentially leading to improved diagnosis and treatment of MIH.

## Conclusion

This study shed light on dentists’ experiences and practices in managing MIH, suggesting that specialisation and diagnostic confidence may influence their perceptions and approaches. While dentists demonstrated basic knowledge and practical experience with MIH, many highlighted the need for further education. The predominant use of composite materials and tooth extractions, however, may reflect a potential bias influenced by the dentists’ age and a possible lack of familiarity with minimally invasive techniques or resin-modified glass ionomer cements. Additional research is necessary to determine the extent to which such factors affect treatment decisions and whether targeted educational interventions could help update knowledge on contemporary treatment options for MIH.

### Recommendations / clinical relevance

It is essential that dentists are adequately trained and educated to diagnose and determine appropriate treatments in a timely manner for each patient. There is a need to develop updated courses that address the aetiology, diagnosis, and management of MIH lesions, based on international guidelines for the clinical management of patients with MIH.

## Data Availability

No datasets were generated or analysed during the current study.
